# Sex-Based Clinical Outcomes Following Percutaneous Closure of Patent Foramen Ovale

**DOI:** 10.3390/jcm15030957

**Published:** 2026-01-24

**Authors:** Giulia Santagostino Baldi, Sebastiano Gili, Giovanni Teruzzi, Giuseppe Calligaris, Piero Montorsi, Daniela Trabattoni

**Affiliations:** 1 Centro Cardiologico Monzino, IRCCS, Via Parea, 4, 20138 Milan, Italy; giulia.santagostino@cardiologicomonzino.it (G.S.B.); sebastiano.gili@cardiologicomonzino.it (S.G.); giovanni.teruzzi@cardiologicomonzino.it (G.T.); giuseppe.calligaris@cardiologicomonzino.it (G.C.); 2 Dipartimento di Scienze Cliniche e di Comunità, Università degli Studi di Milano, 20122 Milan, Italy; piero.montorsi@ccfm.it

**Keywords:** PFO closure, outcomes, sex-based differences

## Abstract

**Objectives:** Although sex differences have been emphasized in stroke and congenital heart disease, there has been limited investigation into their role in patent foramen ovale (PFO) closure for secondary prevention of stroke. We aimed to explore differences by sex in baseline profiles, procedural characteristics, and short-term outcomes of patients undergoing transcatheter PFO closure. **Methods:** A retrospective analysis was conducted on 458 consecutive patients (265 women and 193 men) treated with PFO closure at Centro Cardiologico Monzino in Milan between 2006 and 2011. Baseline information included demographic characteristics, medical history, diagnostic and procedural information, and periprocedural complications. Post-closure outcomes were assessed at index hospitalization and during the first follow-up. **Results:** The indications for PFO closure were as follows: cryptogenic stroke/TIA in 78% of women vs. 88% of men (*p* = 0.04). Positive thrombophilic screening was observed in 16% of women vs. 19% of men (non-significant). We observed age-matched (mean age 44 ± 12 years) patients without sex-related differences in baseline and procedural characteristics, with the exception of greater arterial hypertension in women. The mean follow-up time was 13 years for both groups. Recurrent stroke was observed in 0.1% and TIA observed in 0.4% of the ‘cryptogenic stroke/TIA’ group; in the ‘other indications’ group, 1.4% experienced stroke and no TIA was reported. No significant differences were present between sexes. **Conclusions:** There were no differences in procedural and short-term outcomes between males and females undergoing transcatheter PFO closure, but significant baseline differences in risk factors were identified. There is a critical need for long-term, systematic studies to understand sex and gender differences in the PFO population.

## 1. Introduction

Patent foramen ovale (PFO) is one of the most common congenital heart defects in adults, affecting approximately 25–30% of the population [1]. The presence of PFO has been recognized as a rare cause of paradoxical embolization, potentially causing stroke, especially in young patients [2,3,4].

Randomized controlled trials have demonstrated that percutaneous PFO closure significantly reduces the risk of recurrent ischemic stroke compared with medical therapy alone, leading international guidelines to recommend device closure for secondary prevention in appropriately selected patients [5,6,7,8,9,10,11].

Despite robust evidence supporting the efficacy of PFO closure, sex- and gender-specific aspects of patient selection, procedural risk, and long-term outcomes remain insufficiently characterized. This represents an important knowledge gap, as sex differences are well documented across cardiovascular and cerebrovascular diseases. Women and men differ in stroke epidemiology, vascular biology, thromboembolic risk, bleeding susceptibility, and response to antithrombotic therapies [12,13,14]. In other cardiovascular interventions—such as atrial fibrillation ablation or transcatheter valve therapies—sex-specific differences in outcomes and complications have led to refined risk stratification and management strategies. However, similar systematic evaluation has not been consistently applied to PFO closure.

While at this moment significant data can be found on the demographic, anatomical, and biological factors of patients with PFO and stroke [3], there is sparse information concerning the prevalence of these factors in women and men [4,15,16,17]. Recognizing these limitations, both the Canadian Stroke Best Practice Recommendations [18] and the European position paper on PFO management [19] have emphasized the need for improved sex-specific reporting and analysis in PFO-related research. Such analyses are essential to ensure equitable application of guideline recommendations and to identify potential differences in complication profiles, including atrial fibrillation, device-related events, bleeding, and long-term mortality.

Against this background, we present the results of a single-center observational study aimed at evaluating sex-specific clinical characteristics, procedural features, and long-term outcomes in patients undergoing percutaneous PFO closure for cerebral ischemic events. The extended duration of follow-up provides a unique opportunity to assess the durability of closure efficacy and the potential impact of sex on late complications and survival.

## 2. Methods

This is an observational, retrospective, single-center, and non-profit investigation which enrolled consecutive patients who underwent patent foramen ovale (PFO) closure at the Centro Cardiologico Monzino between January 2006 and December 2011.

The indications for PFO closure were determined according to routine clinical practice and the recommendations available at that time, even prior to randomized clinical trials proving the superiority of PFO closure over standard medical therapy. Indications were classified as a history of stroke, transient ischemic attack (TIA), systemic embolism, or silent cerebral embolism identified by brain magnetic resonance imaging (MRI).

Baseline clinical characteristics were collected, including cardiovascular risk factors and neurological history. Cerebral imaging data were gathered to verify the pattern of ischemic lesions (cortical or subcortical lesions, or negative brain imaging). Other causes of neurological events were excluded by carotid ultrasound, thrombophilic screening, ambulatory ECG monitoring (or loop recorder interrogation), transcranial Doppler, and transthoracic or transesophageal echocardiography. The RoPE (Risk of Paradoxical Embolization) score was calculated for each patient. If atrial fibrillation, significant carotid atherosclerosis (stenosis ≥ 50%), or uncontrolled hypertension were detected during the work-up, patients were not considered suitable for PFO closure.

Procedures were performed under fluoroscopic and transesophageal echocardiography during the earlier period, followed by routine use of intracardiac echocardiography guidance. The choice of the device was at the physician’s discretion.

Procedural details included the type and size of the implanted device, procedure duration, contrast volume (when administered), and radiation exposure. Acute residual shunt was assessed in all patients using standard transthoracic echocardiography the day after the intervention.

Antithrombotic therapy prescribed at discharge included single-antiplatelet therapy (SAPT), dual-antiplatelet therapy (DAPT) with acetylsalicylic acid (ASA) and a P2Y12 inhibitor, or anticoagulation in combination with ASA or a P2Y12 inhibitor. Procedural success and complications were documented.

Follow-up was carried out through phone interviews or on-site visits. Adverse events included death, stroke or TIA, new-onset atrial fibrillation, and bleeding events. The extent of residual shunt on follow-up was also evaluated using contrast-enhanced transthoracic echocardiography.

### Statistical Analysis

Continuous variables were analyzed using means and standard deviation or medians and interquartile ranges; categorical variables were described by numbers and percentages. Baseline and PFO characteristics, procedural data, and follow-up were analyzed using the χ^2^ test for categorical variables and *t* test for continuous variable. Medians were confronted with the Mann–Whitney U test. Two-sided *p* < 0.05 was used for statistical significance.

## 3. Results

### 3.1. Patient Characteristics

The study population is composed of 458 consecutive patients: 193 men and 265 women. Men were slightly older than women (mean age of 47 ± 12 years vs. to 44 ± 12 years, *p* = 0.009).

Cardiovascular risk factors were not particularly represented in the study population, with hyperlipidemia and arterial hypertension being the most frequent; both were more common in men, with arterial hypertension reaching a statistically significant difference (*p* = 0.002), while dyslipidemia showed a trend toward significance (*p* = 0.059). In contrast, the prevalence of diabetes mellitus and current smoking was similar between men and women (Table 1).

Neurological history—including prior TIA, prior stroke, and migraine with or without aura—did not differ significantly between the two groups. Similarly, the findings on brain MRI were evenly distributed, with cortical and subcortical lesions being equally distributed; also, negative brain MRI showed no statistically significant sex-related difference.

### 3.2. PFO Characteristics

Regarding the indication for PFO closure (Table 2), prior TIA and prior stroke were the most prevalent indication in men (87.5 vs. 78.1%, although not statistically significant). Even systemic embolization was comparable in both sexes (*p* = 0.331) as an indication. The only significant difference was represented by a positive brain MRI without corresponding symptoms, which was significantly more frequent in women than in men (*p* = 0.0003).

As for PFO morphology, the prevalence of atrial septal aneurysm did not differ substantially between men and women. The mean RoPE score was higher in women (7.18 vs. 6.88 in men, *p* = 0.013). A history of previous deep vein thrombosis or pulmonary embolism occurred at comparable rates in both sexes.

### 3.3. Procedural Characteristics

The procedural characteristics are summarized in Table 3. All procedures were performed with local anesthesia and with the guide of transesophageal echo in the first period followed by intracardiac echo guidance. All devices were double-disc. The distribution of device types was similar between the two groups. The most frequently implanted device was by far Amplatzer PFO Occluder (Abbott, Chicago, IL, USA); other devices (GSO, Gore; Premere, St. Jude Medical; Ultrasept, Cardia; Figulla Flex, Occlutech) were less often used. Device size selection showed comparable patterns across sexes, with no statistically significant variation in the use of small (≤20 mm), medium (21–25 mm), or large (≥26 mm) devices.

Procedural metrics—including procedural time, contrast, and radiation doses—did not differ between men and women. Procedural success was 100% in both groups (Table 3).

Complication rates were uncommon and did not differ meaningfully by sex. Device embolization occurred only in women (two cases). In-hospital atrial fibrillation was the most frequent complication, but was not different between groups. Post-procedural residual right-to-left shunt was also comparable, with mild and moderate shunts distributed similarly and no severe shunts reported in either group.

Antithrombotic therapy at discharge was not different: most patients received dual-antiplatelet therapy for at least three months, while only a small number of patients received aspirin alone or anticoagulation combined with a single antiplatelet agent.

### 3.4. Follow-Up

Mean follow-up was approximately 14 years overall, with no differences in men and women (Table 4). Residual shunt rates were low in both groups, with mild shunts occurring rarely and no moderate or severe shunts at all. The incidence of new-onset atrial fibrillation was comparable between men and women during the first 1–2 months after PFO closure, despite being underrepresented. All-cause mortality, however, differed significantly, with 14 deaths in men compared with 6 in women (*p* = 0.018), more frequently due to cancer (n = 7), SARS-CoV-2 infection (n = 3), and heart failure (n = 3). No ischemic strokes occurred in either group during follow-up, while transient ischemic attacks were rare, with two cases reported in women only (*p* = 0.576).

Non-life-threatening bleeding events occurred similarly between the two groups (two minor due to epistaxis and three major due to gastrointestinal bleeding). All bleeding events occurred in patients on SAPT except for one in a patient on NOAC treatment for deep vein thrombosis prophylaxis. Given the small number of events, no further analyses were performed.

## 4. Discussion

In this observational cohort of 458 consecutive patients undergoing percutaneous PFO closure, we found that men and women present with broadly similar clinical, anatomical, and procedural profiles, but with specific sex-related differences in selected characteristics and long-term outcomes. Specifically, men were slightly older with a higher burden of traditional cardiovascular risk factors, particularly hypertension and dyslipidemia, while women tended to have a higher risk of paradoxical embolization. These findings are consistent with a study showing an older age at PFO closure for men [20] and with the largest study on sex differences in PFO published to date [6,17]. Unlike the studies by Flores-Umanzor et al. [16] and Farjat-Pasos et al. [17], which reported a higher prevalence of migraine among women, our analysis did not identify any significant sex-related differences in neurological history. These sex-based differences, however, did not translate into differential neurological presentation or MRI lesion patterns apart from silent infarcts.

Women had a significantly higher rate of silent ischemic lesions on MRI, while the prevalence of stroke or TIA was similar in both sexes. Silent lesions may represent either underrecognized prior embolic events or sex-related differences in cerebral perfusion or embolic susceptibility. Prior studies have suggested that women may exhibit a higher propensity for migraine-related microembolization, endothelial dysfunction, or hormonal influences that modify vascular reactivity [21]. Yet the literature on silent infarcts in PFO populations remains sparse.

Despite these differences, procedural success was universal (100%), peri-procedural complications were rare, and long-term prevention of recurrent ischemic stroke was excellent in both sexes, with no strokes observed over a median follow-up of 14 years.

We found no significant sex differences in PFO morphology, including the prevalence of atrial septal aneurysm (ASA). This is consistent with anatomical studies suggesting that PFO dimensions and ASA prevalence do not differ by sex [22]. The RoPE score—which integrates age, risk factors, and imaging features—was slightly higher in women, like the study of Farjat-Pasos et al. [17].

Regarding technical aspects of the procedure, device type and size did not differ significantly between sexes, although there was a trend toward the use of larger devices in men. Procedural factors—including device selection, fluoroscopy time, procedural duration, and complication rates—were comparable between sexes. Universal procedural success highlights the robustness and reproducibility of contemporary PFO closure techniques across diverse patient profiles.

The only two cases of device embolization occurred in women, though the numbers are too small for meaningful interpretations. Prior studies have not shown sex differences in device stability, and both events were successfully managed without long-term sequelae. These outcomes align with contemporary device-era data from large registries and randomized trials [5,6,9,22].

A major strength of this study is the exceptionally long follow-up (mean >14 years), exceeding that of most published randomized trials and registries. The primary goal of PFO closure is to reduce the risk of recurrent cerebrovascular events. Farjat-Pasos et al. [17] reported no sex-related differences in isolated outcomes of stroke or TIA, although composite events (stroke + TIA) were more common in women. Conversely, Flores-Umanzor et al. [16] found no differences in cerebrovascular outcomes between sexes. Similarly, in our study, across the long follow-up period, no ischemic strokes occurred in either sex, confirming the durable benefit of PFO closure in patients selected according to international guidelines [18]. These observations reinforce the long-term real-world evidence on the absence of major sex disparities in procedural efficacy, safety, and neurologic outcomes of PFO closure.

The incidence of new-onset atrial fibrillation remained low and consistent with the 3–6% incidence typically reported in the literature after PFO closure, and not related to the sex [23]. These results suggest that post-procedural atrial arrhythmogenesis is primarily related to mechanical device–septum interaction rather than sex-dependent atrial substrate differences. Additionally, prior studies have shown that AF following PFO closure is often transient and rarely associated with long-term thromboembolic complications [24,25]. The occurrence of atrial fibrillation during follow-up may undermine the protective effect of PFO closure because of the associated cardioembolic risk [26]. All studies [16,17]—including ours—found no sex-related differences in the risk of new-onset atrial fibrillation, thus confirming the overall procedural safety profile.

To our knowledge, three major studies have compared outcomes between sexes. The study by Flores-Umanzor et al. [16], enrolling 783 patients with a median follow-up of 14 years, found no differences in the incidence of cerebrovascular events, new-onset atrial fibrillation, or survival. Two additional studies [4,17], which had shorter follow-up periods, reported similar findings. The same authors demonstrated a higher risk of bleeding among women. Conversely, in our study, bleeding events were rare, non-life-threatening, and non sex-related.

Most studies report that patients are discharged on dual-antiplatelet therapy (DAPT), after percutaneous PFO closure, although findings differ regarding potential sex-based differences in antiplatelet management. In a large multicenter cohort, approximately 61% of patients were discharged with DAPT (typically ≤3 months), whereas others received single-antiplatelet therapy [27]. In another clinical practice analysis, the vast majority (≈91%) of pts were discharged on DAPT for 3–6 months, consistent with recommendations from position papers suggesting DAPT followed by single-antiplatelet therapy [23]. Our study confirms the trend to discharge the majority of patients on DAPT.

## 5. Future Directions

The potential for sex-related differences in outcomes after PFO closure remains an important consideration for individualized patient care. While our results do not confirm major disparities, clinical, procedural, and long-term results, prospective studies with systematic arrhythmia monitoring and extended follow-up are needed to better define the impact of sex on post-PFO closure outcomes.

## 6. Study Limitations

Despite the long follow-up, this study is limited by its retrospective, single-center design and can only be interpreted as hypothesis-generating. The absence of systematic long-term rhythm monitoring may have underestimated AF incidence. Furthermore, our findings may not be generalizable to different practice settings. Despite these limitations, the relatively large cohort and extended observation period strengthen the validity of our observations.

## 7. Conclusions

In this large, single-center, long-term cohort, percutaneous PFO closure proved to be highly effective and safe in both men and women, with no recurrent ischemic strokes observed over more than a decade of follow-up. While baseline characteristics differed by sex—with women more frequently exhibiting silent cerebral ischemic lesions and men demonstrating a higher prevalence of traditional cardiovascular risk factors—these differences did not translate into disparities in procedural success, residual shunt, or long-term neurological outcomes.

These findings provide robust real-world evidence that the protective benefits of PFO closure are equally durable across sexes, affirming the applicability of current guideline recommendations to both men and women. At the same time, the higher prevalence of silent ischemic lesions in women highlights a potentially underrecognized sex-specific substrate that may inform future diagnostic, monitoring, and management strategies.

Ultimately, this study underscores the importance of integrating sex- and gender-based analyses into PFO research and clinical practice. While procedural efficacy appears equivalent, careful attention to sex-specific presentation, referral patterns, and cerebral imaging findings could optimize patient selection, enhance individualized care, and refine long-term follow-up strategies. Future prospective studies with standardized sex-stratified protocols are essential to fully elucidate the biological, anatomical, and clinical nuances of PFO closure across sexes, ensuring that all patients derive maximal benefit from this intervention.

## Figures and Tables

**Table 1:** Baseline characteristics.

	Men (n = 193)	Women (n = 265)	*p*-Value
Age, years (mean ± SD)	47 ± 12	44 ± 12	0.009
**Risk factors**			
Hypertension, n (%)	15 (7.8)	4 (1.5)	0.002
Hyperlipidemia, n (%)	20 (10.4)	14 (5.3)	0.006
Diabetes mellitus, n (%)	1 (0.5)	2 (0.75)	1
Current smoking, n (%)	8 (4.1)	12 (4.5)	1
RoPE score, (mean ± SD)	6.88 ± 1.22	7.18 ± 1.33	0.013
Previous DVT or PE, n (%)	8 (4.1)	10 (3.8)	1
**Neurological history**			
Prior TIA, n (%)	129 (66.8)	163 (61.5)	0.257
Prior stroke, n (%)	40 (20.7)	44 (16.6)	0.301
Migraine, n (%)	56 (29)	81 (30.6)	0.714
Aura, n (%)	11 (5.7)	13 (4.9)	0.864
**Cerebral imaging findings**			
Cortical, n (%)	52 (26.9)	65 (24.5)	0.610
Subcortical, n (%)	46 (23.8)	67 (25.2)	0.830
Negative, n (%)	95 (49.2)	133 (50.2)	0.932

Data are expressed as mean ± standard deviation or as number (percentages). SD, standard deviation; RoPE, risk of paradoxical embolization; DVT, deep vein thrombosis; PE, pulmonary embolism; TIA, transient ischemic attack.

**Table 2:** PFO closure indications and characteristics.

Indication for Closure			
Prior TIA, n (%)	129 (66.8)	163 (61.5)	0.257
Prior stroke, n (%)	40 (20.7)	44 (16.6)	0.301
Systemic embolization, n (%)	11 (5.7)	9 (3.4)	0.331
Positive MRI without symptoms, n (%)	13 (6.8)	49 (18.5)	0.0003
**PFO morphology**			
Atrial septal aneurysm, n (%)	49 (25.4)	59 (22.2)	0.499

Data are expressed as number (percentages). TIA, transient ischemic attack; MRI, magnetic resonance imaging; PFO, patent foramen ovale.

**Table 3:** Procedural characteristics and acute results.

Procedural Variable	Men (n = 193)	Women (n = 265)	*p*-Value
**Device type**			
Amplatzer, n (%)	173 (89.6)	227 (85.7)	0.225
Gore, n (%)	2 (1.0)	4 (1.5)	0.986
Premere, n (%)	7 (3.7)	17 (6.4)	0.272
Other, n (%)	11 (5.7)	17 (6.4)	0.917
**Device size**			
≤20 mm, n (%)	75 (38.9)	127 (47.9)	0.096
21–25 mm, n (%)	69 (49.7)	118 (44.6)	0.341
≥26 mm, n (%)	22 (11.4)	20 (7.5)	0.206
Procedural time, min, median (IQR)	24 (19–34)	24 (18–32)	0.377
Radiation dose, mGy, median, (IQR)	422.5 (230–879)	404 (226–781)	0.358
Procedural success, (%)	100%	100%	n.s.
**Complications**			
Device embolization, n (%)	0 (0)	2 (0.75)	0.625
In-hospital AF, n (%)	11 (5.7)	13 (4.9)	1
Ventricular ectopy, n (%)	0 (0)	1 (0.4)	1
Paroxysmal supraventricular tachycardia, n (%)	2 (1.0)	0 (0)	0.343
**Post-procedural residual right-to-left shunt**			
Mild, n (%)	18 (9.3)	17 (6.4)	0.423
Moderate, n (%)	2 (1.0)	4 (1.5)	0.986
Severe, n (%)	0 (0)	0 (0)	n.s.
**Antithrombotic therapy at discharge**			
ASA, n (%)	0 (0)	1 (0.4)	1
DAPT, n (%)	191 (99.0)	263 (99.2)	1
Anticoagulant + ASA or P2Y12 inhibitor, n (%)	2 (1.0)	1 (0.4)	0.778

Data are expressed as median (interquartile range) or as number (percentages). Other devices include Biostar, Flatstent, Cardia, Occlutech, and Atriasept. AF, atrial fibrillation. ASA, acetylsalicylic acid; DAPT, dual-antiplatelet therapy.

**Table 4:** Follow-up.

Follow-Up Variable	Men (n = 193)	Women (n = 265)	*p*-Value
Length of follow-up (mean ± SD)	14.01 ± 1.91	14.12 ± 1.61	0.525
**Residual shunt**			
Mild, n (%)	7 (3.6)	11 (4.2)	0.975
Moderate, n (%)	0 (0)	0 (0)	n.s.
Severe, n (%)	0 (0)	0 (0)	n.s.
Death, n (%)	14 (7.2)	6 (2.2)	0.018
New-onset AF, n (%)	6 (3.1)	5 (1.9)	0.595
Ischemic stroke, n (%)	0 (0)	0 (0)	1
TIA, n (%)	0 (0)	2 (0.75)	0.576
Bleeding, n (%)	4 (2.7)	1 (0.4)	0.202

Data are expressed as mean ± standard deviation or as number (percentages). AF, atrial fibrillation; TIA, transient ischemic attack.

## Data Availability

The data presented in this study are openly available at https://zenodo.org/uploads/17764485 (doi: 10.5281/zenodo.17764485; accessed on 29 November 2025).
